# Targeted Genome Sequencing (TG-Seq) Approaches to Detect Plant Viruses

**DOI:** 10.3390/v13040583

**Published:** 2021-03-30

**Authors:** Solomon Maina, Linda Zheng, Brendan C. Rodoni

**Affiliations:** 1 Microbial Sciences, Pests & Diseases, Agriculture Victoria, 110 Natimuk Road, Horsham, Victoria 3400, Australia; 2 Australian Grains Genebank, Agriculture Victoria, 110 Natimuk Road, Horsham, Victoria 3400, Australia; 3 Microbial Sciences, Pests & Diseases, Agriculture Victoria, AgriBio, 5 Ring Road, Bundoora, Victoria 3083, Australia; Linda.Zheng@agriculture.vic.gov.au (L.Z.); brendan.rodoni@agriculture.vic.gov.au (B.C.R.); 4 School of Applied Systems Biology (SASB), La Trobe University, Bundoora, Victoria 3083, Australia

**Keywords:** plant virus, crops, genome, diagnostics, high-throughput sequencing

## Abstract

Globally, high-throughput sequencing (HTS) has been used for virus detection in germplasm certification programs. However, sequencing costs have impeded its implementation as a routine diagnostic certification tool. In this study, the targeted genome sequencing (TG-Seq) approach was developed to simultaneously detect multiple (four) viral species of; *Pea early browning virus* (PEBV), *Cucumber mosaic virus* (CMV), *Bean yellow mosaic virus* (BYMV) and *Pea seedborne mosaic virus* (PSbMV). TG-Seq detected all the expected viral amplicons within multiplex PCR (mPCR) reactions. In contrast, the expected PCR amplicons were not detected by gel electrophoresis (GE). For example, for CMV, GE only detected RNA1 and RNA2 while TG-Seq detected all the three RNA components of CMV. In an mPCR to amplify all four viruses, TG-Seq readily detected each virus with more than 732,277 sequence reads mapping to each amplicon. In addition, TG-Seq also detected all four amplicons within a 10^−8^ serial dilution that were not detectable by GE. Our current findings reveal that the TG-Seq approach offers significant potential and is a highly sensitive targeted approach for detecting multiple plant viruses within a given biological sample. This is the first study describing direct HTS of plant virus mPCR products. These findings have major implications for grain germplasm healthy certification programs and biosecurity management in relation to pathogen entry into Australia and elsewhere.

## 1. Introduction

In many parts of the world, plant viruses constitute almost 50% of pathogens causing damaging disease in many agricultural and horticultural crops [1]. Minimizing the impacts of these viral diseases through effective and sustainable disease management is pivotal to reach the 60% increase in food production needed by 2050 [2]. As such, integrating technologically innovative rapid diagnostic tools offers unprecedented breakthroughs in disease management. Studies have been published on applying metagenomics approaches using high-throughput sequencing (HTS) to detect plant viruses [3,4,5,6]. Despite the enormous potential of metagenomics, its greatest challenge is the prohibitive cost associated with using this approach as part of a routine diagnostic strategy. In addition, typical metagenomic sequence datasets are usually predominantly composed of host-derived sequences with only a minor fraction of viral sequences [7], especially if no ribosomal depletion is conducted. Nevertheless, with several modifications HTS holds immense potential and could have a paradigm shift within plant virus diagnostics. Such modifications involve using a targeted universally conserved barcode marker within a taxon for broad spectrum detection. 

A major obstacle to using HTS in a more efficient manner for virus detection is the variable genome structure of RNA viruses [8] as this creates a hurdle in designing diagnostic makers to detect a broad range of species [9]. Unlike other taxa, plant viruses lack a universally conserved barcode that spans multiple genera and families, such as 16S for bacteria, rbcL and matK for plants Cytochrome C oxidase for insects and mammals [10,11,12]. To address this conundrum, multiplex PCR methods have been successfully used to detect multiple viruses simultaneously in a single assay [13]. For example, six to nine plant viruses have been detected simultaneously in one multiplex PCR (mPCR) reaction [14,15,16,17]. This approach reduces costs and turn around diagnostic timelines through amplifying multiple genome regions of virus targets simultaneously. Nevertheless, there are several limitations for mPCR: (i) its sensitivity might be compromised [18]; (ii) use of gel electrophoresis to visualise the presence of an amplicon or discriminate the size of specific amplicons is prone to a putative optical error and is labour intensive [13]; and (iii) lack of subsequent amplicon sequence to confirm the identity of the target viral genome. To address these complexities using HTS, we explore a targeted genome sequencing (TG-Seq) method, which is an amplicon sequencing approach that involves sequencing of an mPCR reaction to detect multiple nucleic acid viral genome targets. Amplicon sequencing [19], shares a similar principle to TG-Seq as it allows targeted analysis of a specific virus genome region, reducing background host sequence data, sequencing costs and downstream bioinformatic analysis complexity in detecting viral sequences [20]. The singleplex and multiplex sequencing approach has been commonly applied in animal and human virus diagnostics [8,21,22]. So far, no studies have demonstrated the application of TG-Seq to detect multiple plant viruses simultaneously. To achieve this, four viruses of major significance to the Australian grain industry were selected: *Cucumber mosaic virus* (CMV; genus *Cucumovirus*, family *Bromoviridae), Pea early browning virus* (PEBV; (genus *Tobravirus,* family *Virgaviridae*)*, Bean yellow mosaic virus* (BYMV) and *Pea seed-borne mosaic virus* (PSbMV); (genus *Potyvirus*, family *Potyviridae*). They were used to investigate the capability of TG-Seq to simultaneously detect these multiple plant virus genome targets and compared its utility with gel visualisation of mPCR products and RNA-Seq approaches. 

## 2. Materials and Methods

### 2.1. Sample Collection

Virus-infected leaf material from faba bean infected with CMV, lentil infected with BYMV, field pea infected with PSbMV were collected in Victoria, Australia, and faba bean infected with PEBV ((isolate Lyv66) imported from Libya) was intercepted at the Australian border in post entry quarantine (PEQ). All virus isolates were desiccated on CaCl_2_ and stored at room temperature.

### 2.2. RNA Extraction and cDNA Synthesis 

The total RNA from the four virus-infected plant samples was extracted using the RNeasy Plant Mini Kit (Qiagen, Hilden, Germany) according to manufacturer’s instructions. Crude RNA was treated with RNase-free DNase (Invitrogen, Carlsbad, CA, USA) and quantified using the Qubit™ RNA BR Assay (Thermo Fisher Scientific, Waltham, MA, USA) then later stored at −80 °C. The isolated total RNA was converted to cDNA using random hexamers and the ImProm-II TM Reverse Transcription System (Promega, Madison, WI, USA). The cDNA was purified using Qiaquick (Qiagen) purification columns and quantified using a Qubit dsDNA high sensitivity assay kit (Thermo Fisher Scientific).

### 2.3. Metagenomics Library Preparation (RNA-Seq)

The RNA-Seq libraries were prepared using a TruSeq stranded Total RNA Sample Preparation kit with Ribo-zero^TM^ Plant (Illumina, San Diego, CA, USA) using RNA templates (BYMV-442 ng/uL, PSbMV-484 ng/uL, CMV-350 ng/uL, PEBV-1674 ng/uL) and each sample was normalized to 1 µg of 10 µL (Supplementary Table S1). Due to the low quality of RNA, the library preparation procedure was slightly modified by omitting the RNA fragmentation step and increasing the volume of Agencourt AMPure XP beads (Beckman Coulter) by 20% in all library purification steps. The Ribozero depleted RNA (s) was primed with random hexamers and converted to double stranded cDNA according to manufacturer’s instructions. Set A adaptors containing the identifier sequences and flow cell binding sequences were ligated to both ends and the cDNA fragment was enriched by 15 PCR cycles, as described by the manufacturer’s instructions (Illumina, San Diego, CA, USA). The integrity of each library was verified using Qubit™ 4 Fluorometer (Invitrogen™, Carlsbad, CA, USA) and D1000 ScreenTape (TapeStation 2200, Agilent Technologies, Santa Clara, CA USA). Each library was diluted to a 10 nM concentration and pooled to achieve an equimolar pooled library concentration. The sequencing of the four pooled denatured libraries was conducted on a MiSeq platform using a v3 kit (Illumina) with 2 × 251 cycles and 1% PhiX v 3 (Illumina) spike was included to generate paired-end reads.

### 2.4. Primer Design and PCR Optimization

A total of 16 primer pairs were designed using OligoArchitect™ (Sigma-Aldrich, St. Louis, MO, USA) from nucleotide genome sequences of PEBV, CMV, BYMV and PSbMV (Table 1) targeting open reading frames (ORF) of PEBV (12K,14K,30K,201K), PSbMV (CP, NIb, HcPro, CI) BYMV (CP, HcPro) and CMV (RNA1, RNA2, RNA3) (Table 2). Total RNA extracts used for library preparation were used as a template for RT-PCR using Superscript™ One-Step RT-PCR System with Platinum™ (Invitrogen™) and cDNA (Supplementary Table S1) was amplified using DreamTaq™ Hot Start Green PCR Master Mix (Thermo Scientific™). Several PCR parameters, such as annealing temperature, extension and cycling times were investigated to determine the optimal combination of the mPCR/RT-PCR assay with each primer having a concentration of 10 pmol/μL in a 20 µL reaction. Two µL each from RNase/DNase- free water and RNA from a healthy oat plant were included as negative controls. Optimum cycling conditions were determined as follows: 50 °C for 30 min for reverse transcription, 95 °C for 15 min followed by 35 cycles at 95 °C for 30 s, 59 °C to 64 °C for 40 s (gradient), and 72 °C for 45 s, with a final extension at 72 °C for 10 min. The amplified specific PCR products generated by the 16 primer pairs (Table 2) were confirmed by gel electrophoresis (GE) followed by SYBR safe staining. An optimum temperature of 62 °C was selected as the best annealing temperature for all subsequent singleplex and mPCR reactions. To confirm the integrity of our optimization, 12 distinct samples each from CMV and PSbMV-infected samples were subjected to the above conditions. RNase/DNase-free water and healthy virus negative oat RNA were included as negative controls. The purified PCR amplicons were sent for Sanger sequencing in Australia in a genome research facility for partial sequencing. 

### 2.5. Singleplex Amplicon PCR and Sequencing

To determine the percentage of reads that are amplicon specific, a singleplex PCR reaction using specific ORF primers (HcPro-1F/HcPro-1FR,PCP-F1/PCP-F1R,201K-F/201K-R,CMVRNA1F/CMVRNA1R) of the four viruses (Table 2), that were tagged with the following Illumina overhang adapter sequences: forward overhang: 5′TCGTCGGCAGCGTCAGATGTGTATAAGAGACAG; and a reverse overhang: 5′GTCTCGTGGGCTCGGAGATGTGTATAAGAGACAG. For each singleplex reaction 2 µL of cDNA (Supplementary Table S1) from each virus was amplified using virus-specific primers tagged with Illumina overhang sequences used at a concentration of 10 pmol/μL in a 20 µL PCR reaction in triplicates using a high-fidelity PCR master mix (Roche). The same negative controls (RNase/DNase-free water and cDNA from healthy oat RNA) were also included. Target amplification conditions were as follows: 95 °C for 3 min, 35 cycles of 94 °C for 40 s, 62 °C for 45 s and 72 °C for 60 s and a final extension at 72 °C for 7 min. Each of the PCR product (18 µL) was cleaned using 40 μL Ampure XP DNA purification beads (Beckman Coulter, Brea, CA, USA) and amplicon quality was verified using GE. The PCR product was ligated with Nextera XT indexes (Illumina) followed by final purification according to the manufacturer’s instructions. The final fragment size and concentration of each library was verified using Qubit™ 4 Fluorometer (Invitrogen™), and the D1000 ScreenTape (Tape Station 2200, Agilent Technologies). Each library was diluted to a 10 nM concentration and pooled to achieve an equimolar pooled library concentration. The run involved sequencing a pool of 12 denatured libraries in a MiSeq using a 2 × 301 v3 (Illumina) kit to generate paired-end reads; a 10% PhiX v 3 (Illumina) spike was included to generate paired-end reads.

### 2.6. Multiplex PCR (mPCR) and Sequencing (Include Negative Control Statement)

**Figure 1:**
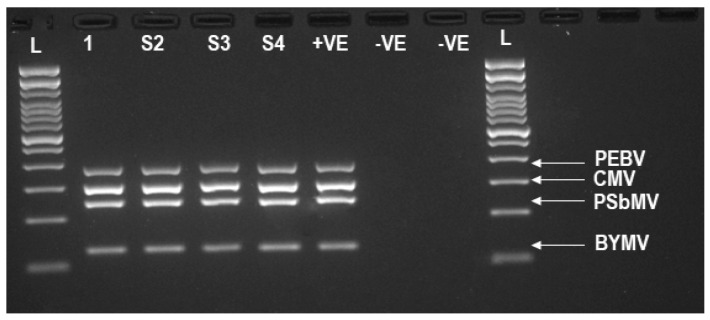
Agarose gel electrophoresis from an mPCR of the four viruses (BYMV, PSbMV, CMV and PEBV) quadruplicate (×4) samples. The (+VE) positive control infected viral RNA pooled together from (BYMV, PSbMV, CMV and PEBV) infected samples amplified using HcPro-1F/HcPro-1FR,PCP-F1/PCP-F1R,CMVRNA1F/CMVRNA1R,201K-F/201K-R primers. The (-VE) negative controls were RNase/DNase-free water and a viral-negative sample (healthy oat plant). L = Invitrogen ready to use 1 kb Plus DNA ladder used on both right and left side of the gel.

mPCR was performed using primer pairs targeting either three or four genome regions in each virus, or targeting a genome region from multiple viruses, in a single PCR reaction (in several cases duplicated or triplicated. Equal mixtures of cDNA from PEBV, CMV, BYMV and PSbMV, was prepared as described above (Supplementary Table S1). The RNase/DNase-free water and healthy oat plant sample 2 µL from each were included as negative controls. Using a high-fidelity PCR master mix (Roche Basel, Switzerland), the mPCR reaction was conducted with 2 µL from each of the four cDNA reactions as template, with each virus specific primer (Table 2) having a concentration of 10 pmol/μL in each PCR reaction with the following cycling conditions: 95 °C for 3 min, 35 cycles of 94 °C for 40 s, 62 °C for 45 s and 72 °C for 60 s and a final extension at 72 °C for 7 min. The amplified mPCR products were confirmed by electrophoresis for 90 min on a 2% agarose gel with SYBR safe staining (Figure 1. Twelve mPCR reactions targeting three to four different viral genomic regions were selected (Table 3). The mPCR products were cleaned using the QIAquick PCR Purification Kit (Qiagen) including the negative controls. The concentration of each cleaned mPCR product was determined using the Qubit™ 4 Fluorometer (Invitrogen™), and the D1000 ScreenTape (Agilent). A volume of 30μL from each of the 12 TG-Seq cleaned PCR products (100–500 ng) was subjected to bead-linked transposomes Nextera DNA flex library preparation kit (Illumina) for amplicon library preparation following the manufacturer instructions. The final amplicon clean-up was done by adding 81 μL of Agencourt Ampure XP DNA purification (beads) (Beckman Coulter) to the amplify tagmented product and the sample mixed by pipetting 10 times. The samples were incubated at room temperature for 5 min, then trapped (DynaMag-2 magnet; Thermo Fisher) using a magnetic stand for 5 min followed by freshly prepared ethanol clean-up as described by the manufacturers protocol. The tubes were then placed on the plate magnet stand for 2 min before 30 μL of the supernatant containing the DNA library was transferred to a fresh tube. The final concentration and fragment size of each library was determined using Qubit™ 4 Fluorometer (Invitrogen™) and high sensitivity D1000 ScreenTape (TapeStation 2200, Agilent Technologies). Each library (Supplementary Table S1) was diluted to a 10 nM concentration and pooled to achieve an equimolar pooled library concentration. The run involved sequencing a pool of 12 denatured libraries in a MiSeq using a 2 × 301 v3 (Illumina) kit to generate paired-end reads and a 1% Phix v 3 (Illumina) spike was included to generate paired-end reads.

### 2.7. Serial Dilutions Multiplex PCR (mPCR) and Sequencing 

An equal mixture of BYMV, CMV, PSbMV and PEBV cDNA was generated by mixing 5 µL each of cDNA together. A 2 µL aliquot from this viral cDNA pool (10^−0^) was used as a template in a 100-fold serial dilution (10^−2^, 10^−4^, 10^−6^, 10^−8^) (Supplementary Table S1). The mPCR was performed using four primer pairs HcPro-1F/1R, PCPF1/R1, 201K-F/R, CMVRNA1F/1R targeting four genome regions of BYMV, PSbMV, PEBV and CMV respectively in replicates (Table 2). RNase/DNase-free water and healthy oat plant DNA were included as negative controls. The TG-Seq PCR reaction was conducted using a high-fidelity PCR master mix (Roche) in a final reaction volume of 44 µL using amplification conditions as follows: 95°C for 3 min, 35 cycles of 94 °C for 40 s, 62 °C for 45 s and 72 °C for 60 s and a final extension at 72 °C for 7 min. The amplified mPCR products were confirmed by 2% agarose gel with SYBR safe (Invitrogen) staining followed by electrophoresis for 90 min (Supplementary Figure S1). The TG-Seq PCR products were cleaned using the QIAquick PCR Purification Kit (Qiagen). A volume of 30 μL of one representative (a sample chosen from each of the dilution series) purified PCR product (100–500 ng) was subjected to Nextera DNA flex library preparation kit (Illumina) following the manufacturer’s instructions, and sequenced as described above.

### 2.8. Sequence Analysis

Quality control of the RNA-Seq raw reads obtained was done using Trim Galore [23]. The RNA-Seq reads were subjected to de novo assembly using the metaSPAdes version 3.13.0 genome assembler [24] with default settings. In addition, a second assembler CLC Genomics Workbench (version 20) (CLCGW) (CLC bio; Qiagen) was used as described^4^ with the minimum contig length set to 800 bp. All the contigs were subjected to BLASTN search using BLAST version 2.7 [25]. The contigs with plant virus matches were used for downstream analysis (Supplementary Table S2). The contigs of interest were imported into Geneious Prime 2020 and aligned using MUSCLE [26]. The ORFs were predicted and annotations made using Geneious Prime 2020 [27], with transfer annotation selected and similarity set at 90%, while other settings were left as defaults. Finalized sequences were designated as complete coding sequences based on comparison with the reference sequences available in public databases (Table 1). 

The TG-Seq derived FASTQ files were first inspected using FastQC (version 2.0) to determine any downstream quality control requirement. Quality control of the TG-Seq raw reads was done using Trim Galore [23] and CLC Genomics (version 20) (CLCGW) (CLC bio; Qiagen). The trimmed TG-Seq reads were subjected to de novo assembly using the metaSPAdes version 3.13.0 genome assembler [24] with default settings to obtain dominant genome sequences. All metaSPAdes-derived contigs were subjected to BLASTN search using BLAST version 2.7 [25] to inspect the expected viral species homology match in each library (Table 3). To determine the deconvoluted genome viral regions reads within a multiplexed TG-Seq library, the genome regions that derived targeted CMV, PEBV, PSbMV, BYMV primers were imported into CLC Genomics package and mapped back to the TG-Seq raw reads using CLC genomics with the following settings; no masking option, followed by reading alignment score match 1, mismatch 2, linear gap cost, insertion and deletion cost 3, length fraction 0.5, similarity fraction 0.8 with automatic detection of paired distances and nonspecific match handling of map randomly to produce stand-alone read mappings (Table 3). In addition, the TG-Seq raw reads were also analysed using the MG-RAST [28] database server to characterize and confirm the viral profile.

## 3. Results

### 3.1. RNA-Seq and TG-Seq Detection of Individual Monopartite, Bipartite and Tripartite Viruses

The local isolates of two monopartite viruses, pea seed-borne mosaic virus (PSbMV) and bean yellow mosaic virus (BYMV); a bipartite virus, pea early browning virus (PEBV); and a tripartite virus; cucumber mosaic virus (CMV) were sequenced (Table 1) and 16 primer pairs were designed to amplify a range of open reading frames (ORFs) of each viral genome (Table 2). Several multiplex reactions were tried using three to four primer pairs targeting a range of ORFs. The monopartite linear genomes of BYMV and PSbMV were each amplified using primer pairs designed from the nuclear inclusion protein (NIb), coat protein (CP), helper component proteinase (HcPro) and cylindrical inclusion (CI) region of the potyviral genome. An mPCR assay was designed to amplify three regions of the BYMV ORF and analysed by gel electrophoresis and by TG-Seq (Table 3; libraries 1–4). The NIb2 (Table 3; library 1) and NIb3 (Table 3; library 2) amplicons were not observable upon gel visualization (Table 3; supplementary Figure S1), while the TG-Seq detected all the amplicons in libraries 1–4, with both library 1 and 2 having 1,208,788 and 22,057 reads mapping on the NIb amplicon respectively. A second mPCR assay targeting four regions of the PSbMV genome was designed and used to amplify PSbMV cDNA. 

Both gel electrophoresis and TG-Seq detected all four amplicons with each amplicon having more than 967,465 reads mapping to it (Table 3; library 5). Two PEBV multiplex reactions were conducted targeting the 12K, 14K, 30K, 201K ORFs of the PEBV genome (Table 3; libraries 6, 7). Both gel electrophoresis and TG-Seq detected all of the expected amplicons with the TG-Seq generating a range of 153,998 to 1,371,956 reads for each amplicon (Table 3). An mPCR assay to detect each of the three RNA components of CMV was optimised. Gel electrophoresis only showed amplicons for RNA1 and RNA2 while TG-Seq detected all three amplicons with amplicon-specific read numbers ranging from 260,700 for RNA3 to 1,293,807 reads for the RNA2 amplicon (Table 3; library 8).

### 3.2. TG-Seq Detection of Multiple Monopartite, Bipartite and Tripartite Viruses in One Assay

To test the sensitivity of TG-Seq for simultaneous detection of multiple genetically diverse plant viruses, a series of mPCR reactions was designed to detect three to four plant viruses in the one assay (Figure 1, Table 3; Libraries 9–12). The initial mPCR involved amplifying the RNA3, 201K and CP of CMV, PEBV and PSbMV respectively (Table 3–Library 9). Visualisation of the bands by gel electrophoresis, as well as TG-Seq detected the amplicon from each virus with more than 732,277 reads mapping to each amplicon. A second mPCR reaction was designed to amplify all four viruses targeting RNA3, 201K, HcPro and CP of CMV, PEBV, BYMV and PSbMV (Table 3 Library 10). RNA3 of CMV was not detected by gel visualisation, whereas all four amplicons were detected by TG-Seq with RNA3 having only 207 reads mapping to it (Table 3; Library 10). Based on the sensitivity of the TG-Seq for CMV RNA3 detection (Table 3, library 10) an additional primer cocktail combination targeting CMV RNA1, as well as 201K, CP and HcPro regions of PEBV, PSbMV and BYMV respectively was tried. This multiplex reaction only detected RNA1 and HcPro when visualized by gel electrophoresis while TG-Seq detected all the ORFs generating 703,928 reads for CMV RNA1, 8929 reads for PEBV (201K), 5348 reads for PSbMV (CP) and 1,057,739 reads for BYMV HcPro (Table 3; Library 11). This mPCR assay was repeated on fresh nucleic acid extracts and all four target ORFs from CMV, PEBV, PSbMV and BYMV were detected by gel electrophoresis and TG-Seq with each amplicon generating more than 645,571 reads (Table 3 library 12; Figure 1). When eight distinct ORFs derived from three diverse viruses (BYMV-CP and HcPro), (PEBV-30K, 141K and 201K) and (CMV-RNA-1–3) were amplified in a single assay followed by library preparation and sequencing, all the amplicons were detected by the TG-Seq approach as follows; PEBV (30K-10.49%,141K-10.23%,201K-10.25%), CMV(RNA1-19.07%, RNA2-2%, RNA3-3.67%), BYMV (CP-31.89%, HcPro-12.03% and nontargets 0.37%.

### 3.3. Sensitivity of TG-Seq in Detecting Serially Diluted Multiple Viruses in One Assay

In order to determine the sensitivity and suitability of the TG-Seq to detect the four viruses simultaneously, a 100-fold serial dilution of cDNA (10^−0^) for each of the four viruses in nuclease free water was generated (10^−2^, 10^−4^, 10^−6^, 10^−8^) and used as a template for mPCR (Table 4). Gel visualisation of the mPCR detected three amplicons (PEBV-201K, CMV-RNA1, BYMV-HcPro) but not PSbMV-CP for the 10^−2^ serial dilution and only detected two amplicons (CMV-RNA1, BYMV-HcPro) for the 10^−4^, 10^−6^, 10^−8^ serial dilutions with the HC-Pro amplicon being nearly invisible in the gel (Supplementary Figure S1, Table 4). However, the TG-Seq detected all the amplicons at each dilution, ranging from only 2224 reads for PSbMV CP amplicon at a 10^−2^ dilution to 1,465,542 reads for CMV RNA1 at the same 10^−2^ dilution (Table 4).

### 3.4. Sensitivity Comparison between RNA-Seq and TG-Seq as Detection Tools

**Figure 2:**
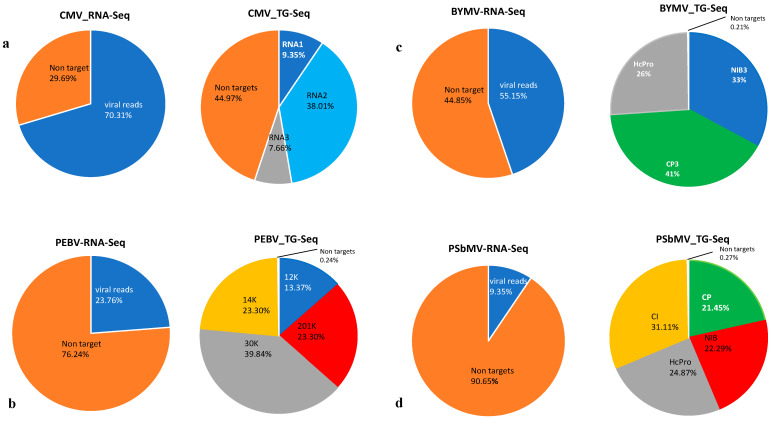
The proportion comparison of RNA-Seq and TG-Seq data based on the number of reads mapping to the virus genome region of interest. (**a**) A comparison of CMV-RNA-Seq and TG-Seq library using specific primer CMVRNA1F/CMVRNA1R for CMV, (**b**) A comparison of PEBV-RNA-Seq and TG-Seq library using PEBV specific primer 201K-F/201K-R, (**c**) A comparison of (BYMV-RNA-Seq and TG-Seq library using BYMV specific primer HcPro-1F/HcPro-1FR, (**d**) A comparison of PSbMV-RNA-Seq and TG-Seq library using PSbMV specific primer PCP-F1/PCP-F1R.

**Figure 3:**
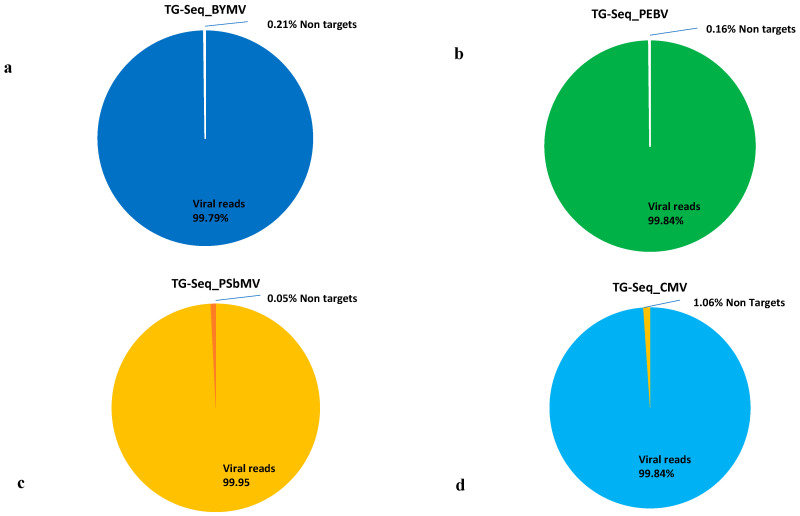
The proportion of number of reads generated through singleplex TG-Seq after mapping back to the region of interest. (**a**) BYMV-TG-Seq library, (**b**) PEBV-TG-Seq library, (**c**) PSbMV- TG-Seq library, (**d**) CMV-TG-Seq library.

When the TG-Seq derived raw reads (from singleplex and multiplex approaches) and RNA-Seq derived raw reads were mapped back to the ORF and the genomes of interest respectively, over 98% mapping reads from the TG-Seq libraries were viral reads, and less than 0.24% of the library reads were nontargets (Figure 2 and Figure 3). On the other hand, the RNA-Seq consisted of up to 90.65% nonviral reads (Figure 2).

## 4. Discussion

This paper describes the first application of a TG-Seq approach to simultaneously detect multiple plant virus genome targets. The TG-Seq proved to be highly sensitive compared to end point singleplex and mPCR and simultaneously detected four plant viruses: CMVPEBV, BYMV and PSbMV. The TG-Seq enabled targeted sequence analysis of viral genomes and reduced background host sequences to less than 1% of total sequenced reads. 

TG-Seq proved to be a highly sensitive and unambiguous detection approach in detecting both individual and mixed genomic regions of PSbMV, BYMV, PEBV and CMV when compared to end point mPCR. For example, three genomic regions of BYMV and CMV were successfully detected by TG-Seq, when compared to two BYMV (NIb2 and NIb3) and one CMV (RNA3) amplicons not being detected by mPCR upon gel visualization. These observations are in line with previous studies that have found the sensitivity of mPCR is influenced by the number of different amplicons generated in a given reaction [31,32,33] Despite the mPCR having the advantage of saving time and cost, its sensitivity of detection of some viruses has been found to be lower than that of singleplex RT-PCR [34,35]. In this study we observed that the sensitivity of mPCR to amplify multiple regions of a monopartite genome (e.g., PSbMV) was comparable while for tripartite genomes (e.g., CMV) the sensitivity of the mPCR was variable. These findings are similar to those reported by [35] who found that the sensitivity of multiplex RT-PCR in comparison to singleplex PCR was slightly lower for tripartite virus genomes. This reveals the sensitivity of TG-Seq detecting expected mono-, bi- and tripartite viral target amplicons in a given multiplex reaction.

Real-time PCR has been widely used for the detection and quantification of the low titre pathogens in a given plant sample [36,37,38]. However, in spite of its robustness, very few studies have adopted the real-time PCR as a multiplex detection assay. This is because of its complexities associated with discriminating between different labelled amplicons during melting curve analysis and overlapping excitations within the fluorescent dyes, which hinders the number of multiple amplicons detected in a multiplex real-time PCR [13]. Although we never performed a real-time mPCR comparison with TG-Seq, our study emulated a hypothetical detection of low titre (pathogens) by serially diluting the cDNA of four RNA viruses. The end-point PCR detected three amplicons (PEBV-201K, CMV-RNA1, BYMV-HcPro) but PSbMV-CP within the 10^−2^ serial dilution was not detected after gel visualisation. For the 10^−4^, 10^−6^ and 10^−8^ serial dilutions, gel electrophoresis only detected two amplicons (CMV-RNA1, BYMV-HcPro) with the HC-Pro amplicon being nearly invisible in the gel. This compares to TG-Seq detecting all four amplicons for each serial dilution, including the 10^−8^ serial dilution, suggesting an increase in sensitivity of TG-Seq of up to 10^−6^ when compared to end-point PCR. The limitations of mPCR followed by gel electrophoresis are widely documented and can be as a result of PCR primers competing for amplicons and reagents thus reducing the yield of some of the final amplicons products [39,40]. The lack of gel electrophoresis to detect some of the ORFs could be due to the presence of very low titre templates, primer biases, amplification errors, reducing the yield of the final mPCR product such that the gel electrophoresis was unable to discriminate their sizes. This limited sensitivity of gel electrophoresis to detect low concentration [35,41] and/or the reported “optical error” associated with gel electrophoresis especially when visualizing very low amplified products [13] might have caused some viruses amplicons not to be picked by gel electrophoresis. The TG-Seq approach sequences the multiple amplicons generated in an mPCR reaction overcoming these limitations and the viral sequences generated from these multiple amplicons provide a further immediate homology confirmation of the present targets 

Although high fidelity DNA polymerase was used in amplifying low concentrated nucleic material (serial diluted library), there was variation in the amplicon datasets (number of reads mapping to the virus specific amplicon), as commonly observed in shotgun HTS [42,43,44,45,46]. This variation is possibly a result of using low quantity input material which in this case was the highly diluted cDNA. Previous studies have discussed HTS data variation to be mainly associated with PCR stochasticity, primer and library preparation biases, phasing and prephasing during the sequencing process [43,44,45,46]. In this study, and particularly for the tripartite genome of CMV, the variability of RNA copy numbers within the virus genome, as reported by [47] might explain why there are more RNA1 reads than RNA3 reads generated by TG-Seq. It can be therefore assumed that these variables contributed to the amplicon reads output variability within each TG-Seq library. The transposomes beads normalization approach allowed successful library preparation across a range of input amounts and template types. For example, the immobilizing the transposomes beads improved coverage uniformity from the low titre templates particularly the 100-fold serial dilution of cDNA from the four viruses (10^−2^, 10^−4^, 10^−6^, 10^−8^). The sequences derived from (serial dilution) transposome beads-based libraries had a good quality precision in virus targets calling and detection of low titre amplicons. These findings corroborate with the previous research of [48] that reported the transposome technology enables fast library preparation and improved coverage uniformity at difficult regions. We therefore recommend future improvement of TG-Seq in plant viruses to focus on incorporating transposome beads-based library chemistry.

Metagenomics approaches such as RNA-Seq are robust diagnostic tools, particularly for virus discovery and whole genome sequencing. This approach has been widely used without requiring prior knowledge of the existing pathogen(s) to identify both viruses and viroids in an infected plant [3,49,50,51]. Its adoption in routine plant virus detection and discovery has been hindered by the high sequencing costs required for testing each sample [4]. The Ribo-Zero chemistry rRNA depletion approach has proved to be successful in reducing the amount of extracted plant rRNA [52,53,54], to enrich the viral detection threshold and data coverage but high depth sequencing will still be required to detect low viral titre agents such as phloem-limited viruses [7]. TG-Seq targeted approach proved robust in enriching targeted viral reads with over 98% representing amplicon reads specific to the target viral genome, and less than 0.24% nontarget sequence reads. This is consistent with previous studies where targeted amplicon sequencing revealed over 90% obtained reads matched targeted public health viruses [21]. On the other hand, the RNA-Seq had a low viral read mapping to a particular virus genome of up to 90.65% non-viral reads. The high nonviral reads from RNA-Seq is well documented in other studies [5,55,56]. Notably, the variations of nonviral targets within the RNA-Seq data for instance within our three hosts (field pea, faba beans and lentil) from 29% to 90.65% could be associated with the genetic variation of rRNA present in each plant species [57]. Admittedly, as mentioned above, RNA-Seq is versatile for entire virome profiling than targeted approaches but its costs still remain prohibitive for routine diagnostic adoption. This highlights the significance of TG-Seq as a detection tool when targeting defined virus species within a given biological sample as it only sequences the targeted region (s) of the viral genome (s) rather than the total nucleic material in a given biological sample. Subsequently, this maximizes the sensitivity of the targeted pathogens, reduces the sequencing costs associated with the detection assay and simplifies downstream bioinformatic analyses. 

Taken together, TG-Seq has offered a broad-range capability of detecting multiple plant viruses due to its high sensitivity. Its approach of using targeted primer panels enhances widespread identification of pathogenic plant viruses across multiple plant samples which in turn reduces the cost compared to whole genome sequencing. Whilst our current study focussed on detecting four viruses simultaneously, we recommend future studies to include more target primer panels targeting DNA viruses and additional divergent lineage RNA viruses. We also propose the incorporation of unique dual indices within TG-Seq library reduces the risk of any indexing crossover from multiplexed samples, increasing accurate diagnostics [58,59]. Moreover, adopting TG-Seq within the low capital cost sequencing platforms such as NovaSeq 6000 (Illumina) system, which has an output >20 billion paired-end reads [60], could offer unprecedented breakthrough in plant virus diagnostics. This is because thousands of TG-Seq libraries can be multiplexed together in a NovaSeq improving the coverage and percentage amplicons recovered, leading to a sensitive, accurate and cost-effective diagnostic tool to support germplasm certification programs, biosecurity investigations and baseline surveillance activities. Its utility has the potential to become a routine HTS plant virus diagnostic tool. 

## Figures and Tables

**Table 1:** RNA-Seq paired-end genome sequence data, including sequence depth coverage, GC content and genome size of the *Bean yellow mosaic virus* (BYMV), *Pea seed-borne mosaic virus* (PSbMV), *Pea early browning virus* (PEBV) and *Cucumber mosaic virus* (CMV) isolates used in this study for primer design and target template for TG-Seq verification.

Sample	Host	Virus	Coverage (x) ^c^	No of Read Counts Mapping to the Virus	GC Content	Genome Size	GenBank Accession
14BY ^a^	Lentil	BYMV	2307	1,331,893	39.4%	9868	LC500882
13C	Field pea	PSbMV	718	507,189	41.5%	9852	SRR13206509
LY-2 ^b^	Faba bean	PEBV-RNA1	3899	235,443	40.7%	7037	LC528622
LY-2 ^b^	Faba bean	PEBV-RNA2	5606	1,252,888	42%	2604	LC528623
14C	Faba bean	CMV-RNA1	8774	318,290	45.3%	3215	SRR13197436
14C	Faba bean	CMV-RNA2	37,144	1,293,807	45.3%	2892	SRR13197436
14C	Faba bean	CMV-RNA3	10,615	260,700	47.1%	2188	SRR13197436

^a^ = Genome sequence of PEBV as reported [29], ^b^ = BYMV as reported [30], 13C and 14C = new PSbMV and CMV genome sequences generated from this study. ^c^ = Average coverage depth across the genome (x) times. The three genomes missed a few nucleotides within the 5′UTR and 3′UTR genome regions but all the coding regions were intact.

**Table 2:** Nucleotide sequence, genome location, amplicon size and optimal annealing temperature of 16 primer pairs used in both singleplex and multiplex PCR reactions.

Primer	Target Virus	Target Genome Region	Amplicon	Primer Sequence (5′-3′)	Amplicon Size (bp)	Optimal Annealing Temperature (Tm; (°C)	Primer Position Binding Site
HcPro-1F a	BYMV	HcPro	HcProF1	CCTTGTGGTCGTATCACTTGTAA	132	64.4	1182–1204
HcPro-1R a				CTGAATGGTGCCTCTGGTAAC		64.9	1412–1432
BYHcProF2	BYMV	HcPro	HcProF2	CCTTGTGGTCGTATCACTTGTAA	251	64.4	1199–1222
BYHcProR2				CTGAATGGTGCCTCTGGTAAC		64.9	1429–1449
BYNIb2F	BYMV	NIb	NIb2	AGAGCAATTCAACCAGAGCATAG	283	64.9	8247–8269
BYNIb2R				CACAAGCACCTCATCAGTCTC		64.9	8505–8525
BYNIb3F	BYMV	NIb	NIb3	TTACAGCCGCACCGATTG	288	64.9	7549–7566
BYNIb3R				CGCATCTCAAGAACAGCATTC		65	7766–7786
BYCPF3	BYMV	CP	CPF3	GAATGGACAATGATGGATGGAGAG	287	65.2	8966–8989
BYCPR3				CTAACTGCTGCCGCCTTC		65	9235–9252
HCPF2	PSbMV	HcPro	HcPro	AGTTAGGCATCTGGCAATAG	359	61.3	2028–2047
HCPR2				AGTCCTTAGCATCCTTCTCA		61.8	2367–2386
CI-1F	PSbMV	CI	CI	TTGCGTGATTCGTCTATGC	296	62.4	5227–5245
CI-1R				TGTGCTATCGTTCTTGTAATTGA		62.3	5500–5522
NIbF3	PSbMV	NIb	NIb	GTGCGTCCAGATTGTGAA	328	61.8	8338–8355
NIbR3				TACTTCTATATGGCTCCTGTTCTA		62	8642–8665
PCP-F1 a	PSbMV	CP	CP	GAACATCAGGAACCATCACA	254	61.7	9005–9024
PCP-R1 a				TTCAATACACCACACCATCAA		60.4	9238–9259
12K2F	PEBV	12K	12K	GAAGTGTGCTGTGTCAAC	294	60.4	6279–6296
12K2R				AAACCGAAATCTATGTCATCTC		60.1	6551–6572
14KF4	PEBV	14K	14K	AGATGTGGACGACTCAGTGAA	254	65	2303–2323
14KR4				CGAAGTTGGCGAAGTGGTT		65.1	2538–2556
30KF	PEBV	30K	30K	TCATCGTAGAAGAGAGACTGTGTT	348	65	5626–5649
30KR				ACCGCAACCGTACCTATCT		64.7	5955–5973
201K-F a	PEBV	201K	201K	GGTTAGAAGTGCTGGAAGTGAA	399	64.4	1621–1642
201K-R a				TCATTGGCTTGCGACTCTC		64.3	2001–2019
CMVRNA1F a	CMV	RNA1	RNA1	CTCCCACGGCGATAAAGG	315	57.56	133–150
CMVRNA1R a				GTGACCCAACTTCCTCCGA		58.94	429–447
CMVRNA2F	CMV	RNA2	RNA2	ATAACMTCCCAGTTCTCACC	260	56.23	1488–1507
CMVRNA2R				TGRAARTCRCACCACCAYTT		57.25	1728–1747
CMVRNA3F	CMV	RNA3	RNA3	GAAATTYGATTCRACYGTGTGGG	202	58.02	1601–1623
CMVRNA3R				CTTNCKCATRTCRCCDATATCAGC		56.98	1779–1802

The 16 primer pairs were designed from BYMV (helper component proteinase (HcPro), nuclear inclusion protein (NIb), and coat protein), PSbMV (HcPro, cylindrical inclusion (CI) protein, NIb and CP), PEBV (12K, 14K, 30K and 201K proteins) and CMV (RNA1, RNA2, RNA3). ^a^ = Primers used in the multiplex and serially diluted mPCR reactions. The target genome region represents the region targeted by the specific primer within the viral genome, product size represents the final expected agarose GE size. Primer binding position represent the primer binding region within the BYMV, PSbMV, CMV and CMV genomes generated in Table 1.

**Table 3:** A comparison between targeted genome sequencing (TG-Seq) and gel electrophoresis (GE) to detect BYMV, PSbMV, PEBV and CMV amplicons generated in a multiplex PCR (mPCR) reaction.

Library	Amplicons Targeted by mPCR ^a^	Raw Reads	No. of Reads after QC (%)	Amplicons Detected by TG-Seq ^b^	Amplicons of BYMV, PSbMV, PEBV and CMV, Detected by GE
1	BYMV (NIb2, CPF3, HcProF2)	3,754,078	97.74%	NIb (1,208,788), CP (1,503,144), HcPro (949,413)	CP, HcPro
2	BYMV (HcProF2, NIb3, CPF3)	3,704,546	98.07%	HcPro (1,748,342), NIb (22,057), CP (1,839,520)	CP, HcPro
3	BYMV (NIb2, CP3, HcProF1)	3,563,538	98.04%	NIb (1,487,879), CP (1,091,068), HcPro (906,692)	HcPro, NIb, CP
4	BYMV (CP, HcProF1, HcProF2)	3,523,172	97.85%	CP (880,456), HcPro (2,490,101)	CP, HcPro
5	PSbMV (CP, NIb, HcPro, CI)	4,568,980	98.71%	CP (967,475), NIb (1,005,493), HcPro (1,121,760), CI (1,403,227)	CP, NIb, HcPro, CI
6	PEBV (12K, 14K, 30K, 201K)	4,110,734	98.46%	12K (706,420), 14K (979,796), 30K (1,371,956), 201K (978,813)	12K, 14K, 30K, 201K
7	PEBV (12K, 14K, 30K, 201K)	3,923,838	98.50%	12K (515,527), 14K (900,724), 30K (153,998), 201K (899,718)	12K, 14K, 30K, 201K
8	CMV (RNA1, RNA2, RNA3)	3,457,376	98.44%	RNA1 (318,290), RNA2 (1,293,807), RNA3 (260,700)	RNA1, RNA2
9	CMV (RNA1), PEBV (201K), PSbMV (CP)	3,257,938	98.21%	RNA1 (1,299,800), 201K (1,145,237), CP (732,277)	RNA1, 201K, CP
10	CMV(RNA3), PEBV (201K2), PSbMV (HcPro), BYMV (CP3)	3,318,404	98.37%	RNA3 (207), 201K (1,561,718), HcPro (419,226, CP (1,248,550)	201K, HcPro, CP3
11	CMV (RNA1), PEBV (201K), PSbMV (CP), BYMV (HcPro)	2,210,396	95.24%	RNA1 (703,928), 201K (8929), CP (5348), HcPro (1,057,739)	RNA1, HcPro
12	CMV (RNA1), PEBV (201K), PSbMV (CP), BYMV (HcPro)	2,514,042	98.54%	RNA1 (735,687), 201K (701,502), CP (645,571), HcPro (739,571)	RNA1, 201K, CP, HcPro

This data was generated using the 16 primers designed in Table 2. The amplicon open reading frames (ORFs) targeted by mPCR are the BYMV and PSbMV each amplified using primer pairs designed from the NIb, CP, HcPro and CI (Libraries 1–5). PEBV multiplex reactions (library 7), targeting the 12K, 14K, 30K, and 201K ORFs of the PEBV genome. CMV multiplex reaction involved the three RNA1-3 components (library 8). Series of mPCR reactions to detect three to four plant viruses Libraries 9–12. ^a^ = Corresponding to virus name and specific primers name (s) listed in Table 2 and used to amplify the ORFs shown in the column named “Amplicons detected by TG-Seq^b^”, ^b^ = figures in parenthesis are the number of reads mapping to each genome region of interest.

**Table 4:** A comparison of the sensitivity of targeted genome sequencing (TG-Seq) and gel electrophoresis (GE) to detect BYMV, PSbMV, PEBV and CMV amplicons generated in a multiplex PCR (mPCR) reaction.

Library	Virus	mPCR Product Concentration	Raw Reads	No. of Reads after QC (%)	Virus Amplicons Detected by TG-Seq	Amplicons Detected by GE
10^−^^2^	CMV,PEBV,PSbMV,BYMV	16.9 ng/uL	2,245,566	98.05%	RNA1 (1,465,542), 201K (130,757), CP (2,224), HcPro (519,838)	RNA1, 201K, HcPro
10^−4^	CMV,PEBV,PSbMV,BYMV	8 ng/uL	2,332,290	97.49%	RNA1 (1,831,035), 201K (91,395), CP (27,503), HcPro (239,682)	RNA1, HcPro
10^−6^	CMV,PEBV,PSbMV,BYMV	8 ng/uL	1,924,302	96.71%	RNA1 (807,712), 201K (74,276), CP (246,891), HcPro (724,792)	RNA1 *, HcPro *
10^−8^	CMV,PEBV,PSbMV,BYMV	7 ng/uL	2,221,416	96.45%	RNA1 (1,096,272), (201K) 127,154, CP (210, 534), HcPro (704,258)	RNA1 *, HcPro *

A 100-fold serial dilution (10^−2^, 10^−4^, 10^−6^, 10^−8^) of viral cDNA in nuclease free water from each of the four viruses was used as template. Amplicons detected by TG-Seq, CMV (RNA1), PEBV (201K), PSbMV (CP), BYMV (HcPro). Amplicons detected by gel visualisation at serial dilution 10^−2^ * (201K, RNA1, HcPro) 10^−4^ (RNA1, HcPro), 10^−6^ (RNA1, HcPro *), 10^−8^ (RNA1, HcPro *) = (nearly invisible), () = figures in parenthesis are the number of reads mapping to each genome region of interest.

## Data Availability

All the raw data from both TG-Seq and RNA-Seq were deposited in NCBI Sequence Read Archive under Bioproject accession number PRJNA680190. https://www.ncbi.nlm.nih.gov/sra/PRJNA680190.

## Supplementary Materials

The following are available online at https://www.mdpi.com/article/10.3390/v13040583/s1, Supplementary Figure S1: Agarose gel electrophoresis from a multiplex PCR of the four viruses (BYMV, PSbMV, CMV and PEBV) (2 × 4) reactions. The aliquot from this viral cDNA pool was used as a template in a 100-fold serial dilution (S1,S2 = 10^−2^, S3,S4 = 10^−4^, S5,S6 = 10^−6^, S7,S8 = 10^−8^) infected viral RNA pooled together from (BYMV, PSbMV, CMV and PEBV) infected samples amplified using HcPro-1F/HcPro-1FR,PCP-F1/PCP-F1R,CMVRNA1F/CMVRNA1R,201K-F/201K-R primers, L = Invitrogen ready to use 1 kb Plus DNA ladder. Supplementary Table S1: Summary of concentrations in ng/uL RNA templates used for RNA-Seq, cDNA amount used for PCR and mPCR and finally the library template used for TG-Seq. Supplementary Table S2: Summary of RNA-Seq paired-end data of the four samples LY-2 = Genome sequence of PEBV as reported [29], 14BY = BYMV infected sequenced sample as reported in [30], 13C and 14C = new PSbMV and CMV sequences generated from this study. Percentage of viral reads = number of reads that mapped back to the viral genome of interest.
