# Supplementary material for: Targeted Genome Sequencing (TG-Seq) Approaches to Detect Plant Viruses
Source: Viruses. 2021 Mar 30;13(4):583. doi: 10.3390/v13040583 (PMC8066983; doi:10.3390/v13040583)
Supplement: Supplementary file 1 [file viruses-13-00583-s001.pdf]

Supplementary Table S1. Summary of concentrations in ng/ul RNA templates used for RNA-Seq, cDNA amount used for PCR and mPCR and finally the library template used for TG-Seq.

Samples	Virus	Host	RNA Amount in ng/µL (Nanodrop)	cDNA Amount in ng/µL (Qubit™ 4 Fluorometer)	PCR Product ng/µL (Qubit™4 Fluorometer)	Final Library Amount ng/µL (Qubit™ 4 Fluorometer)
1 (14C)	CMV	Faba bean	350	159	58.2	17.1
2 (13C)	PSbMV	Field pea	484	145	57.6	18.6
3 (LY-2)	PEBV	Faba bean	1674	163	126	22.1
4	CMV, PEBV,PSbMV,BYMV	Pool	354	168	42.7	15.0
5 (LY-2)	PEBV	Faba bean	1674	163	47.7	18.4
6v(14BY)	BYMV	Lentil	442	134	59.5	15.2
7 (14BY)	BYMV	Lentil	442	134	58	14.0
8	CMV, PEBV, PSbMV, BYMV	Pool	354	168	125	14.4
9 (14BY)	BYMV	Lentil	442	134	46.7	14.4
10	CMV, PEBV, PSbMV, BYMV	Pool	354	168	110	6.94
11	BYMV	Lentil	442	134	79	14.4
12	CMV, PEBV, PSbMV, BYMV	Pool	354	168	39.7	12.6
10^{-2}	CMV, PEBV, PSbMV, BYMV	Pool	354	9.6	16.9	54/pg/µL
10^{-4}	CMV, PEBV, PSbMV, BYMV	Pool	354	7.6	8	10pg/µL
10^{-6}	CMV, PEBV, PSbMV, BYMV	Pool	354	6.4	8	10pg/µL
10^{-8}	CMV, PEBV, PSbMV, BYMV	Pool	354	4.3	7	10pg/µL

Pea early browning virus (PEBV), *Cumber mosaic virus* (CMV), *Bean yellow mosaic virus* (BYMV), *Pea seed-borne mosaic virus* (PSbM)

Supplementary Table S2. Summary of RNA-Seq paired-end data of the four samples LY-2=Genome sequence of PEBV as reported Maina et al.2020a, 14BY= BYMV infected sequenced sample as reported in Maina et al. 2020b, 13C and 14C= new PSbMV and CMV sequences generated from this study. Percentage of viral reads= Number of reads that mapped back to the viral genome of interest.

Sample	Host	Raw Reads	No. of Reads After QC	Percentage of Viral Reads	De Novo Assembly Contigs
13C	Faba bean	4,829,138	4,814,173	70.31%	101
14BY	Lentil	3,588,734	3,541,904	55.15%	12,171
LY-2	Faba bean	6,263,484	6,080,125	23.76%	244
14C	Field pea	5,425,232	5,354,502	9.35%	10,396

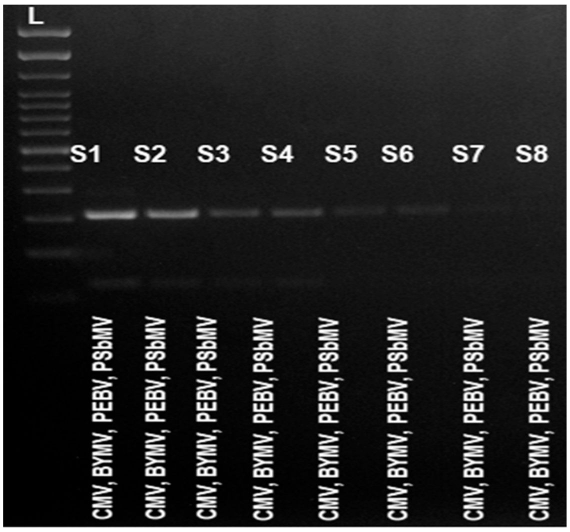

Supplementary Figure S1. Agarose GE from a mPCR of the four viruses (BYMV, PSbMV, CMV and PEBV) replicated (2 × 4) reactions. The aliquot from this viral cDNA pool was used as a template in a 100-fold serial dilution (S1,S2 = 10^{-2}, S3,S4 = 10^{-4}, S5,S6 = 10^{-6}, S7,S8 = 10^{-8}) infected viral RNA pooled together from (BYMV, PSbMV, CMV and PEBV) infected samples amplified using HcPro-1F/HcPro-1FR,PCP-F1/ PCP-F1R,CMVRNA1F/CMVRNA1R,201K-F/201K-R primers, L = Invitrogen ready to use 1 kb Plus DNA ladder.
